# The complete plastid genome sequence of *Welwitschia mirabilis*: an unusually compact plastome with accelerated divergence rates

**DOI:** 10.1186/1471-2148-8-130

**Published:** 2008-05-01

**Authors:** Skip R McCoy, Jennifer V Kuehl, Jeffrey L Boore, Linda A Raubeson

**Affiliations:** 1 Biological Sciences, Central Washington University, Ellensburg, WA 98926-7537, USA; 2 DOE Joint Genome Institute and Lawrence Berkeley National Laboratory, Program in Evolutionary Genomics, Walnut Creek, CA 94547, USA; 3 Department of Integrative Biology, University of California, Berkeley, CA 94702, USA; 4 Genome Project Solutions, 1024 Promenade Street, Hercules, CA 94547, USA

## Abstract

**Background:**

*Welwitschia mirabilis *is the only extant member of the family Welwitschiaceae, one of three lineages of gnetophytes, an enigmatic group of gymnosperms variously allied with flowering plants or conifers. Limited sequence data and rapid divergence rates have precluded consensus on the evolutionary placement of gnetophytes based on molecular characters. Here we report on the first complete gnetophyte chloroplast genome sequence, from *Welwitschia mirabilis*, as well as analyses on divergence rates of protein-coding genes, comparisons of gene content and order, and phylogenetic implications.

**Results:**

The chloroplast genome of *Welwitschia mirabilis *[GenBank: EU342371] is comprised of 119,726 base pairs and exhibits large and small single copy regions and two copies of the large inverted repeat (IR). Only 101 unique gene species are encoded. The *Welwitschia *plastome is the most compact photosynthetic land plant plastome sequenced to date; 66% of the sequence codes for product. The genome also exhibits a slightly expanded IR, a minimum of 9 inversions that modify gene order, and 19 genes that are lost or present as pseudogenes. Phylogenetic analyses, including one representative of each extant seed plant lineage and based on 57 concatenated protein-coding sequences, place *Welwitschia *at the base of all seed plants (distance, maximum parsimony) or as the sister to *Pinus *(the only conifer representative) in a monophyletic gymnosperm clade (maximum likelihood, bayesian). Relative rate tests on these gene sequences show the *Welwitschia *sequences to be evolving at faster rates than other seed plants. For these genes individually, a comparison of average pairwise distances indicates that relative divergence in *Welwitschia *ranges from amounts about equal to other seed plants to amounts almost three times greater than the average for non-gnetophyte seed plants.

**Conclusion:**

Although the basic organization of the *Welwitschia *plastome is typical, its compactness, gene content and high nucleotide divergence rates are atypical. The current lack of additional conifer plastome sequences precludes any discrimination between the gnetifer and gnepine hypotheses of seed plant relationships. However, both phylogenetic analyses and shared genome features identified here are consistent with either of the hypotheses that link gnetophytes with conifers, but are inconsistent with the anthophyte hypothesis.

## Background

*Welwitschia mirabilis *Hook f. (Welwitschiaceae) is a morphologically unique gymnosperm ("without parallel among all living vascular plants" [1]) of the Namib Desert of southwestern Africa (Namibia and Angola) and is the only extant member of its genus and family. The species is dioecious, and each adult plant consists of a giant taproot, a very short woody stem, and two permanent 'strap-shaped' leaves [1,2]. Welwitschiaceae is one of three families in the Gnetophyta (each family would be placed in its own order and class when the group is recognized at the level of phylum), the other two being Ephedraceae, comprised of the genus *Ephedra*, and Gnetaceae, comprised of *Gnetum*. Welwitschiaceae diverged from other gnetophytes prior to the early Cretaceous [3].

Gnetophytes are of intense interest not only due to their peculiar morphology but also because controversies regarding seed plant phylogeny revolve around their placement (reviewed in [4,5]). In morphologically based cladistic analyses [e.g., [6,7]], gnetophytes are the extant sister to angiosperms (the "anthophyte" hypothesis). However the anthophyte hypothesis has rarely been recovered in analyses based on molecular data [4,5,8]. In recent work, multiple outcomes are often supported, even within the same paper, depending on which genes, which sites and which methods are employed [e.g., [5,9,10]]. Gnetophytes have been placed sister to all other seed plants [e.g., [11]], sister to conifers (the "gnetifer" hypothesis) [e.g., [12]], or nesting within conifers as the sister to Pinaceae (the "gne-pine" hypothesis) [e.g., [13-15]].

Placement of the gnetophytes based on molecular data has been problematic, apparently due to accelerated rates of evolution in the lineage, which can lead to Long Branch Attraction or LBA [16,17]. Increased taxon sampling to break up long branches is commonly regarded as an effective approach for overcoming LBA, as is adding more sequence data, selecting slower markers, selecting slower positions and representing lineages with slowly evolving exemplars [18]. Furthermore, using genomic characters, e.g., inversions and gene or intron losses, which are less vulnerable to LBA, can also be helpful [19,20]. In plants, the chloroplast genome is the primary target when attempting to generate large amounts of sequence data and genomic characters for phylogenetics, but unfortunately gymnosperms are very poorly represented among currently available chloroplast genome sequences. This paucity of gymnosperm chloroplast genome sequences (only three gymnosperm plastomes are available, and two are species from the same genus: *Pinus thunbergii *[21], *P. koraiensis*, and *Cycas taitungensis *[22]) currently limits the ability to construct genome level data matrices for seed plant phylogenetics.

The typical seed plant plastid genome [23] contains two copies of a large inverted repeat (IR) separated by large (LSC) and small (SSC) single copy regions. The genome is usually comprised of 150,000 to 160,000 base pairs (bp), includes approximately 120 different genes and is highly conserved in both gene order and content [24-26]. This general form (excepting some minor variation in IR boundaries and some differences in gene content between gymnosperms and angiosperms) is found in plastomes of *Cycas *[22], early-diverging angiosperms (such as *Amborella *[27] and *Nuphar *[28]), magnollids [29,30] and various eudicots [31].

In contrast, some seed plant lineages contain plastid genomes that vary from this typical form [25]. Some plant groups contain genomes that lack one copy of the large IR [32,33] or have greatly expanded IR regions [34]. Gene loss can occur, and the most extreme examples can be found in the plastid genomes of parasitic angiosperms [35]. Rearrangements have affected the gene order of plastid genomes, in some lineages slightly [e.g., [36-38]] but in a few cases greatly [34,39-41]. And, although the rate of evolutionary change of plastid genes is largely conservative [42,43], it is elevated in some lineages [44,45]. Here, in describing the completely sequenced *Welwitschia mirabilis *plastome, we add to these examples of atypical plastid genomes and discuss phylogenetic implications.

## Results

### General Characteristics of the Genome

**Figure 1:**
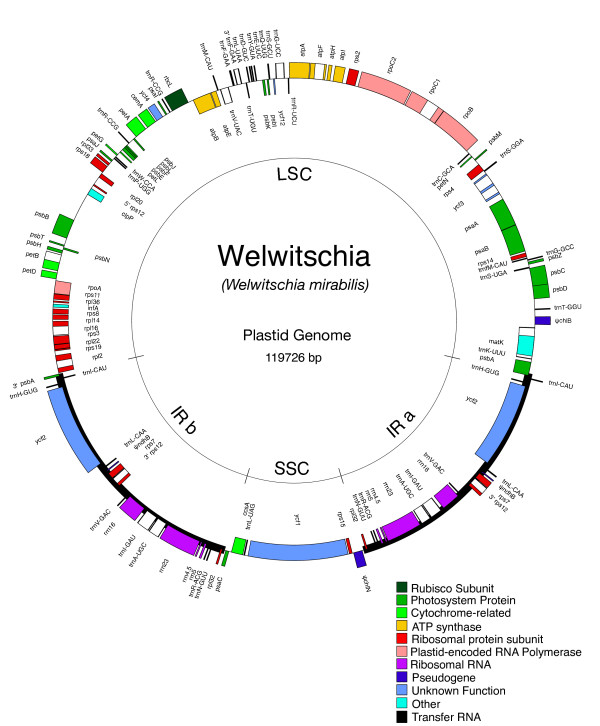
**Gene map of the *Welwitschia mirabilis *plastome.** Genes shown on the inside of the circle are transcribed counterclockwise and those on the outside clockwise. Gene boxes are color coded by functional group as shown in the key in the figure. The genome shows a structure typical to most chloroplast genomes: two copies of the inverted repeat region separating the large and small single copy regions. There are, however, multiple gene-order changes and gene losses relative to the ancestral genome organization, as well as slight expansions of the IR at the LSC and SSC boundaries.

The 119,726 bp *Welwitschia mirabilis *plastid genome [GenBank: EU342371], similar to other chloroplast genomes, is A+T rich overall and in all compartments except for the RNA genes [see Additional file 1 and Additional file 2 for details]. The plastome consists of a large single copy region (LSC) of 68,556 base pairs (bp) and a small single copy region (SSC) of 11,156 bp, separated by two copies of the large inverted repeat (IRa and IRb) of 20,007 bp each (Fig 1). This quadripartite structure is typical among most land plant and some algal chloroplast genomes [46,47]. The small size of the genome is unexpected; *Welwitschia *possesses the smallest plastid genome of any published non-parasitic land plant that still contains the large IR. The genome is similar in size to two other publicly available chloroplast genomes from gymnosperms, *Pinus koraiensis *(116,866 bp) and *Pinus thunbergii *(119,707). However, both *Pinus *plastomes have lost all but 475 or 495 bp (respectively) of their IR [48]. The *Welwitschia *plastome is less than 75% the size of the plastid genome reported for *Cycas taitungensis *[22].

The *Welwitschia *plastome is also unusually compact (i.e., a higher proportion of the genome is comprised of coding sequence and less of non-coding sequence). Coding regions constitute 66% of the *Welwitschia *plastome, making this the most compact of any non-parasitic, land plant chloroplast genome sequenced so far. *Pinus thunbergii *is the most similar, with 61% coding (Table 1). However, the NCBI value for *Pinus thunbergii *(and perhaps some of the other genomes) includes non-conserved ORFs and pseudogenes as coding sequence, which we excluded from coding in our calculations for *Welwitschia*. The NCBI coding percentage values are automatically generated based on the submitted annotation. Therefore, different methods of annotation (e.g., whether or not all ORFs are annotated) lead to ambiguities. For example, we recalculated compactness for the *Pinus thunbergii *plastid genome, using the same approach we applied to the *Welwitschia *genome, and found *P. thunbergii *to be 57.3% coding (rather than the 61% reported at NCBI). Thus our conclusions are conservative, and in no instance would the values reported in Table 1 be increased if calculated using the method applied to *Welwitschia*. Therefore, the compactness of our genome may be even more remarkable when taking into account that the genomes in the NCBI database may be less compact, by our methods of calculation, than reported. Even so, overall, the average coding percentage of the available land plant genomes, using NCBI values and excluding *Welwitschia*, is only 50.0% (Table 1), compared to 66% in *Welwitschia*.

**Table 1:** Compactness measures for vascular plant plastid genomes.

Species	% Coding	Length (bp)	Species	% Coding	Length (bp)
***Welwitschia mirabilis***	**66%**	119,726	*Nicotiana tabacum*	49%	155,943
*Pinus thunbergii*	61%	119,707	*Populus alba*	49%	156,505
*Psilotum nudum*	55%	138,829	*Liriodendron tulipifera*	49%	159,866
*Nicotiana tomentosiformis*	54%	155,745	*Eucalyptus globules*	49%	160,286
*Nicotiana sylvestris*	54%	155,941	*Acorus calamus*	49%	153,821
*Adiantum capillus-veneris*	53%	150,568	*Drimys granadensis*	49%	160,604
*Pinus koraiensis*	52%	116,866	*Jasminum nudiflorum*	49%	165,121
*Oenothera elata*	52%	163,935	*Morus indica*	49%	158,484
*Arabidopsis thaliana*	51%	154,478	*Citrus sinensis*	49%	160,129
*Calycanthus floridus*	51%	153,337	*Liriodendron tulipifera*	49%	159,886
*Helianthus annuus*	51%	151,104	*Daucus carota*	49%	155,911
*Solanum bulbocastanum*	51%	155,371	*Gossypium hirsutum*	48%	160,301
*Lycopersicon esculentum*	51%	155,460	*Vitis vinifera*	48%	160,928
*Lotus japonicus*	51%	150,519	*Spinacia oleracea*	48%	150,725
*Atropa belladonna*	51%	156,687	*Oryza sativa*	48%	134,525
*Panax ginseng*	50%	156,318	*Amborella trichopoda*	48%	162,686
*Solanum tuberosum*	50%	155,298	*Platanus occidentalis*	48%	161,791
*Nymphaea alba*	50%	159,930	*Zea mays*	47%	140,384
*Glycine max*	50%	152,218	*Saccharum officinarum*	47%	141,182
*Coffea arabica*	50%	155,189	*Lactuca sativa*	47%	152,765
*Nandina domestica*	50%	156,599	*Piper cenocladum*	46%	160,624
*Cucumis sativus*	49%	155,293	*Triticum aestivum*	44%	134,545
*Oryza nivara*	49%	134,494	*Phalaenopsis aphrodite*	44%	148,964
*Daucus carota*	49%	155,911	**Non-gnetophyte average**	**50%**	

Percent coding values for species other than *Welwitschia *were obtained from the NCBI database. The average reported in the table was calculated excluding *Welwitschia*.

The LSC boundary of the IR in *Welwitschia *is located in the 3' end of *psbA*. The LSC-end of the IR includes the genes *ycf2*-*trnH*-*trnI*-3'*psbA*, with *ycf2*, *trnH-GUG*, and *psbA *on one strand and *trnI*-CAU on the other. Two events are required to explain the expanded IR seen in *Welwitschia*, from the ancestral seed plant condition (i.e., *ycf2 *and *trnH *duplicated in the IR ancestrally) proposed by Wu et al [22]. First, *trnI-*CAU was duplicated into the IR via an expansion of IR_b _at the J_LB _boundary. Second, IR_a _expanded at the J_LA _boundary to include a portion of *psbA*. Interestingly, the remnant IR found in *Pinus thunbergii*, which includes *trnI *and 3'*psbA*, matches the LSC end of the IR in *Welwitschia *(almost exactly: in the *Welwitschia *plastome only 77 bp more of *psbA *is duplicated). The duplication of *trnI *in this context and followed by a partial duplication of *psbA *is found in no other completely sequenced chloroplast genome, and other methods (targeted PCR and sequencing) show this "motif" to exist only in plastomes of gnetophytes and conifers [22,49]. Presumably the loss of the IR in pines (and perhaps all conifers) is a further modification of a *Welwitschia*-like IR.

**Figure 2:**
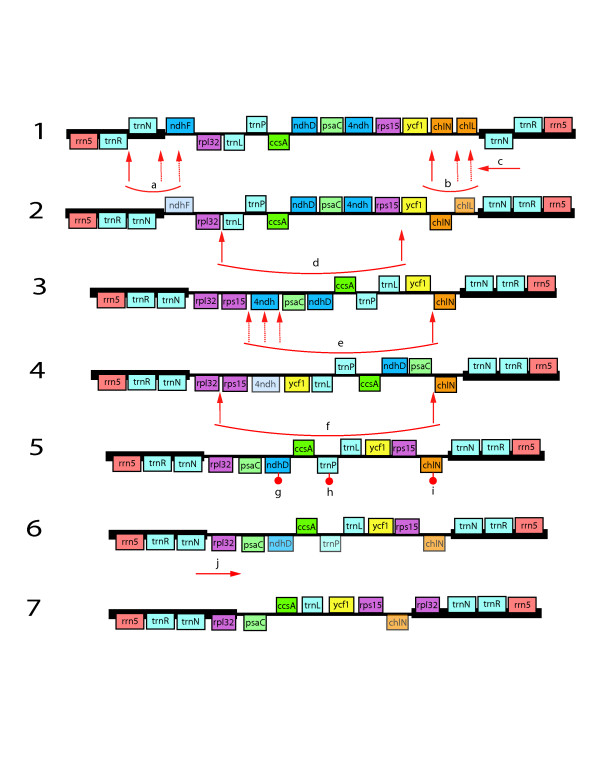
**Rearrangement scenario showing one possible explanation of differences observed in the *Welwitschia *SSC region.** Gene order and orientation are represented in the diagram, but genes and IGS are not shown to scale. Map 1 shows the SSC and flanking ends of the IR as the region appears in *Cycas *or *Ginkgo*, maps 2–6 are hypothetical, and map 7 illustrates the region as it appears in the *Welwitschia *genome. Event a is an inversion that reverses the orientation of *trnN*. One endpoint of this inversion may have disrupted *ndhF*, leading to its loss. Event b is an inversion that reverses *chlN*. One endpoint may have disrupted *chlL*. Event c is the copy correction of the second IR copy to reflect the gene order change (event a) in the other copy. Events d, e, and f are inversions modifying the order and orientation of blocks of genes within the SSC. Again, in step e, inversion breakpoints may have disrupted genes. Events g, h, and i are additional gene losses (*chlN *is still detectable as a pseudogene) not directly related to inversion breakpoints. Event j is an extension of the inverted repeat into the SSC to copy *rpl32 *into the IR. The positions of the inversion endpoints are defined by the gene adjacencies in *Welwitschia *as compared to the ancestral condition, however exactly how those endpoints are combined into inversion events is speculative. Thus the endpoints indicated for events b-f may have been combined in different ways and the events may have occurred in an alternative order than that represented in this model.

The gene order in the *Welwitschia *chloroplast genome is rearranged compared to more "typical" seed plant plastomes such as *Cycas, Amborella*, or *Nicotiana*. There are 14 locations (i.e., breakpoints) where gene adjacencies differ in *Welwitschia *compared to *Cycas taitungensis*, excluding differences due to gene losses and IR boundary shifts. A minimum of nine inversions (i.e., reversals on the chromosomal scale that change the order and orientation of one or more genes) would be required to convert the *Cycas *(ancestral) gene order to that of *Welwitschia *(derived), in addition to gene losses and IR extent changes. No gene order changes occur in the IR, but 4 inversions (7 breakpoints) are proposed for the LSC and 5 inversions (7 breakpoints) for the SSC (Fig 2). Although both *Pinus *and *Welwitschia *plastomes have undergone inversions, they share none in common. Over the entire plastome, there is only one clear instance where both genomes are disrupted at the same general location (*trnT-GGU *and *trnE-UUC*, immediately adjacent in unrearranged land plant plastomes, have been moved apart in both genomes). However, a shared inversion would require two shared points of disruption. There is a second possible shared disruption in the region of *ndhF*. However, even if there is truly a shared point of disruption (hard to define due to gene losses), these two endpoints (*trnT*-*trnE *and *ndhF*) could not have been used together in a single inversion; if these two breakpoints flanked one inversion in *Welwitschia *(with one endpoint in the LSC and the other in the SSC) the IR would have become a direct rather than inverted repeat. Since the repeat is still inverted we can be confident that such an inversion did not take place.

### Gene Content

**Figure 3:**
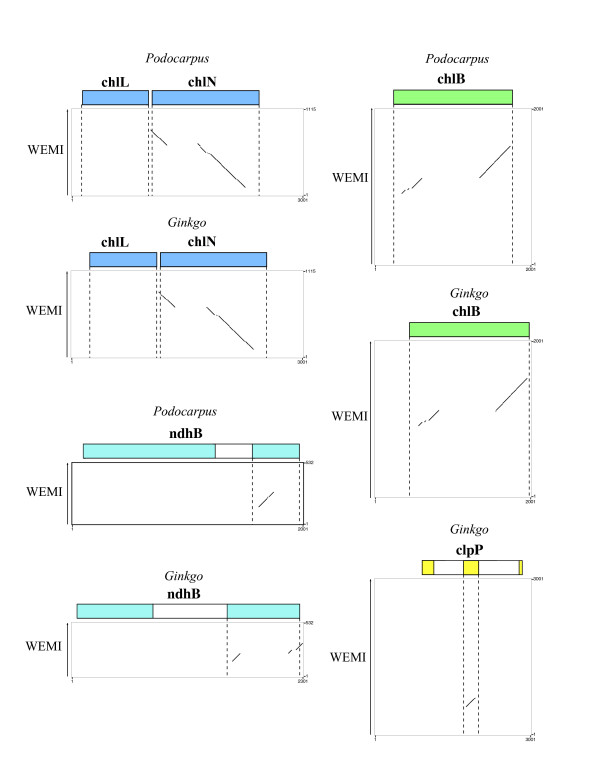
**Harr plots comparing sequence from the *Welwitschia *plastome with homologous regions from *Podocarpus *and *Ginkgo*.** The extent of genes found in each region of the *Podocarpus *and *Ginkgo *plastomes are shown at the top of each graph as colored boxes. The *Welwitschia *(WEMI) sequence is represented as the Y-axis. Diagonal lines indicate regions of similarity between the two sequences being compared. Most portions of these genes found in *Podocarpus *and *Ginkgo *lack equivalent sequence in *Welwitschia *and the sections exhibiting similarity are disjointed.

The *Welwitschia *genome contains 101 distinct, presumably functional, genes: 31 unique tRNA genes, four rRNA gene species, and 66 different protein-coding genes (including 5 widely conserved ORFs or *ycf *genes). Four rRNA genes, eight tRNA genes and four protein-coding genes are fully or in part duplicated in the IR. Of the 18 genes usually found to contain introns in land plant plastid genomes, only 12 are still present in the *Welwitschia *plastome and two, *petD *and *clpP*, lack introns. In addition to genes duplicated in the IR, *Welwitschia *also has a duplication of the *trnR*-CCG gene, as well as a partial duplication of *trnF-*GAA. Of the genes normally present in land plant chloroplast genomes, four (Fig 3) are pseudogenes (detectable but truncated and containing various frameshift mutations resulting in numerous premature stop codons) and 15 are completely absent (at least we were unable to detect any remnants of them in the genome).

The entire complement of *ndh *genes (11 subunits encoding NADH dehydrogenase) is non-functional, with 10 being absent and one, *ndhB*, being a pseudogene. The *ndhB *remnant is a small section of the 5' exon (Fig 3), only 121 bp long, whereas the fully functional *Nicotiana tabacum ndhB *gene is 1,530 bp long. The loss of the *ndh *genes also has been reported in *Pinus *[21], where more of the genes (seven) remain detectable as pseudogenes. In *Welwitschia *elevated rates and selection for compactness could accelerate the loss of detectable gene remnants following the initial loss of function. Presumably the remnant *ndhB *gene is still recognizable because it resides in the IR where rates are reduced [50]. Within completely sequenced land plant plastomes, the *ndh *genes have also been lost from the chloroplast genome of the orchid *Phalaenopsis *[45] and the non-photosynthetic parasite *Epifagus *[51]. The genes are found in some but not all green algal plastid genomes, and are lacking from plastomes of the red algal lineage and *Cyanophora *[52].

The genes for the three *chl *subunits, encoding the enzyme protochlorophyllide reductase, are also missing or non-functional, with one gene (*chlL*) being completely absent and the other two (*chlN *and *chlB*) being pseudogenes. The *chlN *and *chlB *pseudogenes are truncated, to 697 bp and 604 bp respectively, from a full length (as represented by *Pinus thunbergii*) of 1,530 bp and 1,401 bp. Based on earlier work [53], we expected *chlL *to be missing or highly divergent in *Welwitschia*. However results of that previous study, using Southern hybridization, indicated that *chlL *is present in *Ephedra *and *Gnetum*, conflicting with the results of Wu et al [22] for *Gnetum*. An examination of the *Welwitschia *plastome suggests that the loss of the *chl *genes may have been initiated by an inversion. One breakpoint may have been located within the *chlL *gene itself (Fig 2), so that the inversion split the gene apart, disrupting it and causing it to become non-functional. Of course, it is also possible that the gene was inactivated by simple base substitution. Once the *chlL *(or other) subunit was inactivated, no functional enzyme could be produced, eliminating any selection to retain the other two subunits as intact genes. It seems suggestive that the *chlL *gene is completely missing whereas *chlN *and *chlB *remain as pseudogenes, consistent with the hypothesis that *chlL *was lost first. These three genes are also missing from all angiosperm plastomes [31] as well as from the *Psilotum *chloroplast genome [53]. The product of these *chl *genes encodes an enzyme that allows chlorophyll to "green in the dark". An alternative, nuclear-encoded protein that requires light can also affect chlorophyll maturation. Thus the loss of these genes presumably would not be lethal even if the plastid genes were not successfully transferred to the nuclear genome.

Beyond the loss of the *ndh *and *chl *gene families, we were also unable to detect the genes *accD, psaM, rpl23*, *rps16 *or *trnP-GGG *in the *Welwitschia *plastid genome. The gene *accD *has been lost independently in numerous prokaryote and eukaryote lineages [41]. The gene *rpl23 *has been reported missing from the plastome of the angiosperms *Spinacia *[54,55] and *Trachelium *[31]. The gene *rps16 *has experienced numerous independent losses in land plants [31,56,57]. Overall, the gene content of *Welwitschia *appears to be very close to what Wu et al [22] report for *Gnetum*, except that *rps15*, which is reported as absent in *Gnetum*, is present in *Welwitschia*.

### Divergence for Protein-Coding Genes

In most cases *Welwitschia *plastid genes are more divergent than the genes of other seed plants. We attempted to measure relative divergence with a ratio derived from a simple comparison of pairwise distances, an extension of the approach used by Hajibabaei, Xia, and Drouin [58]. For each of 57 genes (Table 2), we calculated the average pairwise distance among non-gnetophyte seed plants and the average pairwise distance between *Welwitschia *and each representative non-gnetophyte seed plant. We then determined the ratio of these two averages to determine a "Relative Divergence Factor" for each gene. If the Relative Divergence Factor is less than one, then that gene in the *Welwitschia *plastome shows less divergence than average, if it is one, the gene is equally divergent, and, if greater than one, the *Welwitschia *gene exhibits above average divergence.

**Table 2:** Comparison of Relative Divergence Factors calculated using different reference sets of non-gnetophyte seed plants.

Gene	setA	set B	set C	set D	set E	average
**All**	**1.69**	**1.53**	**1.67**	**1.50**	**1.54**	1.59
**atpA**	**1.53**	**1.36**	**1.44**	**1.30**	**1.32**	1.39
**atpB**	**1.57**	**1.44**	**1.59**	**1.37**	**1.38**	1.47
**atpE**	**1.59**	**1.52**	**1.63**	**1.44**	**1.47**	1.53
**atpF**	**1.58**	**1.32**	**1.39**	**1.30**	**1.33**	1.38
atpH	1.40	1.36	1.23	1.42	1.38	1.36
**atpI**	**1.60**	**1.56**	**1.64**	**1.45**	**1.50**	1.55
**ccsA**	**1.68**	**1.54**	**1.81**	**1.49**	**1.58**	1.62
**cemA**	**1.51**	**1.27**	**1.51**	**1.22**	**1.27**	1.36
**matK**	**1.92**	**1.77**	**1.91**	**1.72**	**1.81**	1.83
**petA**	**1.64**	**1.48**	**1.68**	**1.43**	**1.49**	1.54
**petB**	**1.74**	**1.79**	**1.83**	**1.66**	**1.74**	1.75
**petD**	**1.67**	**1.52**	**1.65**	**1.54**	1.60	1.60
petG	1.81	1.55	1.95	1.49	1.65	1.69
petN	1.34	1.16	1.16	1.08	1.10	1.17
**psaA**	**1.95**	**1.35**	**1.52**	**1.37**	**1.38**	1.51
**psaB**	**1.53**	**1.33**	**1.42**	**1.36**	**1.37**	1.40
**psaC**	**1.80**	**1.86**	**1.96**	**1.77**	**1.95**	1.87
**psaI**	**2.54**	2.03	**2.27**	**2.02**	**1.95**	2.16
psaJ	1.13	0.87	0.93	1.05	0.88	0.97
**psbA**	**1.51**	**1.32**	**1.38**	**1.29**	**1.34**	1.37
**psbB**	**1.62**	**1.57**	**1.66**	**1.49**	**1.53**	1.57
**psbC**	**1.64**	**1.51**	**1.50**	**1.52**	**1.51**	1.54
**psbD**	**1.76**	**1.68**	**1.67**	**1.46**	**1.66**	1.64
psbE	**1.55**	1.39	**1.74**	1.25	1.35	1.46
**psbF**	**2.97**	**2.64**	**3.45**	**2.97**	**2.67**	2.94
psbH	**1.65**	**1.61**	**1.57**	1.39	1.44	1.53
psbI	1.37	1.45	1.43	1.23	1.34	1.36
psbJ	**2.17**	**2.05**	**2.14**	1.78	1.86	2.00
psbK	**1.54**	1.30	1.35	1.25	1.29	1.34
psbL	1.31	0.97	1.08	1.19	1.03	1.12
psbM	1.33	1.07	1.36	1.02	0.99	1.15
psbN	1.78	1.32	1.50	1.29	1.33	1.45
psbT	1.25	1.39	1.75	0.94	1.16	1.30
**psbZ**	**2.04**	**2.02**	**2.19**	**1.91**	**2.01**	2.03
**rbcL**	**1.48**	**1.42**	**1.61**	**1.32**	**1.36**	1.44
**rpl14**	**1.86**	**1.62**	**1.62**	**1.64**	**1.68**	1.68
**rpl16**	**2.14**	**2.27**	**2.28**	**2.02**	**2.09**	2.16
**rpl20**	**1.61**	**1.53**	**1.54**	**1.45**	**1.53**	1.53
**rpl33**	**2.02**	**2.39**	**2.32**	**2.43**	**2.46**	2.32
**rpl36**	**2.26**	1.99	**2.28**	**2.10**	**1.83**	2.09
**rpoA**	**2.15**	**2.27**	**2.40**	**1.93**	**2.03**	2.15
**rpoB**	**1.89**	**1.69**	**1.82**	**1.49**	**1.58**	1.69
**rpoC1**	**2.07**	**1.69**	**1.77**	1.14	**1.57**	1.65
**rpoC2**	**1.73**	**1.58**	**1.70**	**1.46**	**1.54**	1.60
**rps11**	**2.62**	**2.59**	**2.84**	**2.24**	**2.40**	2.54
rps12	**1.98**	1.72	1.46	**1.49**	**1.60**	1.65
**rps14**	**2.02**	**2.01**	**2.35**	**1.78**	**1.91**	2.01
**rps15**	**1.88**	**1.72**	**1.43**	**1.61**	**1.70**	1.67
**rps18**	**2.99**	**2.15**	**2.43**	**2.40**	**2.11**	2.41
**rps19**	**1.95**	**2.19**	**2.07**	**2.01**	**2.05**	2.05
**rps2**	**2.10**	**2.02**	**2.04**	**1.82**	**1.85**	1.97
**rps3**	**2.18**	**2.20**	**2.35**	**2.02**	**2.10**	2.17
**rps4**	**1.83**	**1.73**	**1.76**	**1.68**	**1.62**	1.72
**rps7**	**2.20**	**2.37**	**2.58**	**1.64**	**1.69**	2.10
rps8	**1.57**	1.29	**1.40**	1.23	**1.32**	1.36
**ycf3**	**1.95**	**1.53**	**1.54**	**1.69**	**1.63**	1.67
**ycf4**	**1.63**	**1.36**	**1.49**	**1.32**	1.05	1.37

Relative divergence factor values indicated in bold are significantly different than 1 (two-tailed t-test. p < 0.05). Detailed information on each set of calculations can be found in Additional files 3, 4, 5, 6, 7.

**Figure 4:**
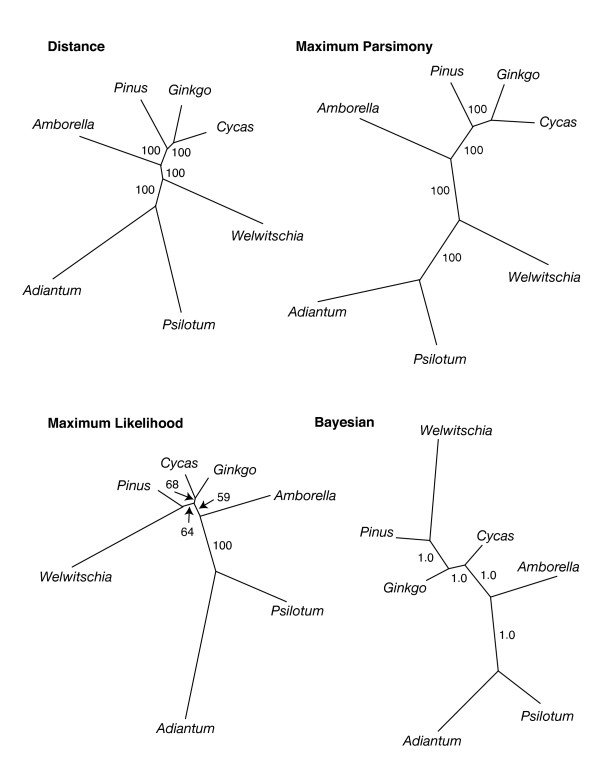
**Phylogenetic hypotheses obtained using different optimization criteria based on 57 protein-coding plastid genes.** Values associated with branches indicate the level of bootstrap support or, for the Bayesian analysis, posterior probability. Branch lengths are shown proportional to inferred amount of change in all trees.

We selected from among 10 taxa to calculate the non-gnetophyte average: *Ginkgo, Cycas, Pinus, Podocarpus, Amborella, Nuphar, Nymphaea, Calycanthus, Ranunculus*, and *Acorus*. We used A) all 10 taxa, as well as B) *Ginkgo *and *Cycas *plus *Ranunculus *and *Pinus*, C) *Ginkgo *and *Cycas *plus *Amborella *and *Pinus*, D) *Ginkgo *and *Cycas *plus *Amborella *and *Podocarpus*, and E) *Ginkgo *and *Cycas *plus *Amborella*, *Pinus *and *Podocarpus*. Phylogenetic structure within the non-gnetophyte taxa could confound this approach. For example, in Set A, which contains multiple angiosperms, *Nuphar *and *Nymphaea*, especially, are rather closely related. Having such "small" distances included in the non-gnetophyte average could bias the result. However, we did not obtain very different results when using single angiosperm exemplars. In terms of the higher-level structure in the non-gnetophytes, internal branches are probably so short (Fig 4) relative to the terminals that this structure would have minimal impact. Also, if the true phylogenetic position of *Welwitschia *is anywhere within the other seed plants, then the bias should be against the *Welwitschia *to non-gnetophyte distances. In summary, this seems a reasonable enough way to investigate which genes are more or less divergent.

Across all the comparisons and all genes, the Relative Divergence Factor ranged from 0.87 to 3.45 (Table 2). [See Additional files 3, 4, 5, 6, 7 for details of the calculations for each reference set.] For about 20% of the comparisons, the difference between the non-gnetophyte average and the *Welwitschia *to non-gnetophtye average was not significant, either because the averages were basically equal or, more often, because the variation around the average was great enough to make them statistically indistinguishable. About 25% of the genes had an average Relative Divergence Factor above 2. When the Relative Divergence Factor was determined as an average of the five different calculations, only one gene (*psaJ*) had a divergence rate about equal to the non-gnetophytes (factor of 0.97), whereas 38 genes had Relative Divergence Factors above 1.5 and 14 above 2.

The average Relative Divergence Factor over all genes for each reference set ranged from 1.50 to 1.69 (all significant, p < 0.0001). The average Relative Divergence Factor from all genes, over all calculations, is 1.68. For all the genes together, we also applied Tajima's Relative Rate Test (Table 3). The *Welwitschia *genes have very significantly (p < 0.00001) elevated rates based on any of the comparisons conducted, whether the three taxon statement was compatible with commonly produced trees or not. In these tests, the proportion of unique sites identified in *Welwitschia *relative to the comparison taxa suggests that the Relative Divergence Factor method might be conservative.

**Table 3:** Tajima's Relative Rate Tests between *Welwitschia *and various other seed plants.

(A, B) C	(WEMI, *Pinus*) *Ginkgo*	(WEMI, *Ginkgo*) *Pinus*	(WEMI, *Amborella*) *Ginkgo*	(WEMI, *Ginkgo*) *Amborella*	(WEMI, *Amborella*) *Pinus*	(WEMI, *Pinus*) *Amborella*
Identical sites	28782	28782	27333	27333	26970	26970
Divergent sites, all 3	718	718	1047	1047	1214	1214
Unique differences Seq A	5456	5456	5088	5088	4488	4488
Unique differences Seq B	1615	1805	3220	1955	3497	2057
Unique differences Seq C (reference)	1805	1615	1955	3220	2057	3497
χ^2 ^statistic	2086.45	1835.81	420.1	1393.68	122.99	902.94
P value (1 df)	0.00000	0.00000	0.00000	0.00000	0.00000	0.00000

### Phylogenetic Inference

Using the same 57 genes (47,858 aligned nt) as for our Relative Divergence Factor and Relative Rate comparisons, we inferred phylogenetic trees using various methods. Here, we also included genes from the pteridophytes *Adiantum *and *Psilotum*. The trees shown (Fig 4) include only *Amborella *as the representative angiosperm. However, analyses using other angiosperms (from those listed as "set A", above) as single exemplars or including all six angiosperm taxa yielded identical topologies (data not shown; however we will note that when the six angiosperms were included together, *Amborella *was strongly supported as the earliest diverging angiosperm by all methods). Neighbor-joining, Minimum Evolution (ME) and Maximum Parsimony (MP) trees yielded topologies with *Welwitschia *diverging first among the seed plants. In contrast, Maximum Likelihood (ML) and Bayesian (BI) analyses resulted in topologies that included a monophyletic gymnosperm clade and a *Welwitschia-Pinus *sister group. Bootstrap (BS) values and posterior probabilities (PP) indicate that each method, except ML, very strongly supports its particular solution. However, the SH test indicates that, for these data, the three topologies shown in Fig 4 do not have significantly different likelihoods. In contrast, the best ML topology (shown in the figure) is significantly better than the best anthophyte topology (p < 0.001).

It is interesting to note that although taxon sampling in our phylogenetic analyses is very limited, the trees obtained are mostly robust (e.g., high BS, PP values) and consistent with expectation. In other studies (e.g., [58] and [5] along with studies reviewed therein), based on total evidence from fewer genes but more taxa, distance methods and MP commonly place gnetophytes at the base of all seed plants, whereas ML and BI support a topology including gnetifers or gnepines. Here we see exactly the same pattern. By all methods, *Welwitschia *is inferred to represent a long branch, consistent with the relative rate tests and the relative divergence factor analyses. Since ME and MP are more likely to be confounded by rate heterogeneity, we would guess that the placement of *Welwitschia *in these trees is an artifact. However, when MP analyses were conducted on nucleotide data with the third position excluded or on deduced amino acid data, both of which should reduce the effects of rate heterogeneity, the same MP topology is recovered. As mentioned previously, according to SH test results, these data (for these taxa) cannot discriminate among the three topologies of Fig. 4. Although the addition of more sequence data allows us (with a sampling of only five seed plant taxa) to do no better than earlier work based on better taxon sampling, likewise our results are no worse. This suggests that a significant increase in number of genes (of a level available from whole plastome sequences) will allow a rather limited taxon sampling to provide important insights into seed plant phylogeny. Of course that sampling will need to be less limited that what is available to us and must include at least one conifer II representative.

## Discussion

The morphology of *Welwitschia *makes it a very unusual organism. The plastid genome also has unusual features. Although the overall organization of the genome is typical of most land plants (i.e., having two single copy regions separated by two inverted repeat regions), the extent of the IR, gene content, gene order, rate of nucleotide divergence, and compactness, all are atypical. The phylogenetic significance of any of these atypical features can, at this point, be interpreted in only a limited manner. Until more gnetophyte plastome sequences are available we are uncertain whether features seen in *Welwitschia *are unique to that species or are characteristic of gnetophytes. Likewise, traits of the *Pinus *plastid genomes provide little detail about the nature of conifer genomes in general. Derived characters shared between *Pinus *and *Welwitschia *are compatible with either the gnetifer or the gne-pine hypotheses; however, they are inconsistent with the anthophyte hypothesis.

There are some differences in gene content that are shared between *Welwitschia *and angiosperms. Angiosperms and *Welwitschia *(but not *Ephedra*) lack the three *chl *genes. Angiosperms and *Welwitschia *(but not *Gnetum*) share the loss of *trnP-GGG*. These loss events could be shared due to common ancestry only if the gnetophytes were not monophyletic, in conflict with almost all recent work. Plastome gene loss has been a common pattern over the history of the plastid as an endosymbiont [52,59]. The same genes are often lost independently in unrelated lineages [52]. Even within angiosperms, where gene content is largely stable in photosynthetic representatives, some genes have been lost in multiple instances (reviewed recently in [31]).

The most distinctive structural feature common to both *Welwitschia *and *Pinus *is the shared IR extent. The *Pinus *remnant IR (a 495 bp sequence containing *trnI *and a 3' portion of *psbA *[48]) matches the LSC end of the IR in *Welwitschia *and *Gnetum *[22] suggesting that the *Pinus *IR represents a reduction from a *Welwitschia/Gnetum*-like ancestor. *Welwitschia *and *Pinus *plastomes also both lack functional copies of all eleven *ndh *genes, the *rps16 *gene, and both introns of *clpP*. However, due to the reasons mentioned above, gene losses must be used cautiously as phylogenetic markers. Phylogenetic analyses, using ML and BI, based on 57 plastid genes also link *Welwitschia *and *Pinus *(as analyses based on 56 genes linked *Gnetum *and *Pinus *[22]). Of course, a representative of the second (non-Pinaceae) lineage of conifers (i.e., "conifer II" or Cupressophyta [60]) is necessary to distinguish between the gnetifer and gne-pine hypotheses.

Based on the *Welwitschia *plastome gene organization, we can speculate (due to placement of breakpoints) that inversions in the LSC or the SSC (Fig 2) may have initially destroyed some *ndh *genes leading to the loss of all the subunits, as was also a possible explanation in the case of the *chlL *gene loss. Inversion endpoints are located in the areas of the *ndhCKJ *cluster in the LSC, as well as *ndhF *and the *ndhHAIGE *cluster in the SSC. If any one of the *ndh *genes was disabled by an inversion (or by any other type of mutation) the remaining subunits would gradually decay to extinction. A gene-disrupting inversion in *ndhF *also may have initiated *ndh *gene loss in Pinaceae. Although an initial disruption of *ndhF *is a viable hypothesis to explain *ndh *loss in both *Pinus *and *Welwitschia*, the gene disrupting inversions could not be held in common between the gnetophyte and Pinaceae plastomes because, as discussed earlier, none of the inversions can be shared. Of course the initial gene disruptions could have been point mutations, not inversions; in which case, the losses of the *ndh *genes could possibly represent a synapomorphy supporting the gnepine clade (as *ndh *genes are reported missing from other gnetophytes [22,61] and yet are commonly amplified from members of Cupressophyta [61]). Unfortunately gene losses lack complex characteristics to aid in determining homology.

We detected elevated levels of sequence divergence in most *Welwitschia *genes analyzed. Earlier work, based on limited numbers of genes [15,62-64], has consistently found gnetophytes to have higher rates of sequence evolution in genes from each of the three compartments (plastid, mitochondrial and nuclear genomes) and in both ribosomal and protein-coding genes. Hajibabaei, Xia, and Drouin [58] showed, for nine genes (four plastid, three nuclear, and two mitochondrial), that the average pairwise distances between gnetophytes and non-gnetophyes were significantly higher than average pairwise distances among the non-gnetophytes. Here we expanded their approach to 57 plastid genes and found that almost all these genes exhibit above average divergence. We also found that, using the Relative Rate Test on the 57 genes, rates in *Welwitschia *were significantly higher in a variety of comparisons. Rates in *Gnetum *also were analyzed [22] using relative rate tests and, although transition rates in *Gnetum *were not always significantly different, transversion rates were. Thus these expanded studies (ours and Wu et al [22]) indicate that plastome-wide rate elevation has probably taken place in the gnetophtyes. Rate elevation is also suggested by the phylogenetic analyses, both in the estimated branch lengths and, presumably, in the conflict in outcomes among the different optimization methods.

The *Welwitschia *chloroplast genome was found to be unusually compact. The compactness of the genome can be interpreted in numerous ways, but might suggest that the small compact *Welwitschia *genome is the result of selective pressure to more rapidly replicate the genome by reducing intergenic space and by losing "non-essential" nucleotides. It is interesting to note that increased rate of replication is one factor hypothesized to lead to increased mutation rate [65]. The small genome size can be attributed to the high incidence of gene loss in addition to compactness. This suggests that a small, compact genome was more important to the success of *Welwitschia *(or, more likely, its ancestors) than the function of any genes lost.

## Conclusion

Here we describe the first completely sequenced plastid genome of a gnetophyte. The *Welwitschia *plastome provides insight into the rates of sequence evolution of this highly divergent group of plants, as well as illustrating the possible gnetophyte pattern of gene loss and rearrangement. The most distinctive, potentially phylogenetically informative, feature of the *Welwitschia *chloroplast genome, the IR extent, supports a relationship of gnetophytes with conifers while being inconsistent with the anthophyte hypothesis. Phylogenetic hypotheses supported in analyses of 57 plastid genes, but minimal taxonomic representation, also lack any support for an anthophyte clade. The availability of the *Welwitschia mirabilis *plastome will provide important information for use in further phylogenetic studies resolving major questions about the evolution of seed plants. As plastome sequences for gymnosperms accumulate, genome level phylogenetic analyses should contribute to the resolution of controversies of seed plant phylogeny; currently however the number of gymnosperm plastomes is very limited. Additionally, further gnetophyte plastomes need to be sequenced in order to determine whether atypical characteristics seen in *Welwitschia *are shared by all gnetophytes, or are unusual genomic features of a very unique plant.

## Methods

### Chloroplast Extraction and DNA Sequencing

Chloroplasts were extracted from leaf tissue of *Welwitschia mirabilis *using the sucrose gradient method as described in Jansen et al [66]. Genomic RCA product (prepared using the Operon Repli-G Kit) was used to prepare the template for shotgun sequencing. Shotgun sequencing was performed at DOE Joint Genome Institute [67]. Assembly and its assessment were conducted using Phrap as implemented in Consed 15.1 [68]. Additional targeted sequencing was conducted on PCR products to attain quality in the finished sequence of a level of Q50 or higher for every nucleotide [69]. Each of the four IR-SC boundaries was also confirmed with independent PCR and sequencing reactions.

### Annotation

The *Welwitschia *genome was annotated with the aid of DOGMA (Dual Organellar GenoMe Annotator) [70,71]. Each region in which genes were not detected using DOGMA was investigated using TBLASTX [72] and ORFfinder [73]. In all cases gene boundaries (start and stop codons as well as intron/exon boundaries) were determined through comparison with other plastome annotations rather than via experimental evidence.

### Coding Percentages

The coding percentage of the *Welwitschia *chloroplast genome sequence was calculated as the proportion of nucleotides that would be represented in mature gene products relative to the total number of nucleotides in the genome. Introns and pseudogenes were not included in the coding percentage calculation. The percent coding of all other genomes reported in Table 1 were obtained from the NCBI database.

### Pseudogenes

Pseudogenes were detected and their extent determined using a variety of approaches. Any region that was found using DOGMA to contain fragmentary sequences of genes was aligned (using ClustalW) to functional versions of genes from various previously sequenced plants. In addition, Harr or dot plots were constructed using Pipmaker [74], under default stringency settings, to compare potentially homologous regions of the *Welwitschia *genome with gene sequences from the plastomes of *Podocarpus macrophyllus *and *Ginkgo biloba *(Raubeson et al, unpublished). The Harr plots were visually analyzed in order to determine presence of significant sequence similarity. Pseudogenes were annotated as the most extensive region of detectable similarity.

### Gene Order and Loss

Gene order rearrangements were investigated manually and using GRIMM [75,76]. When using GRIMM (which requires gene content to be identical), genomes were simplified such that IR expansions or contractions and gene losses were not taken into account. GRIMM reports the minimum number of inversions required to "convert" one genome into another and returns one model of inversions. The specific inversion scenario suggested by GRIMM is only one of several possible models of equal length that could explain the observed pattern. As GRIMM is unable to take into account IR expansion and contraction, visual analyses were also performed in order to take into account these events. These visual analyses were also used to hypothesize the loss of the *ndh *and *chl *genes.

### Divergence

For 57 shared protein-coding genes (these are the 61 plastid genes first used for phylogenetic inference by Goremykin et al [27], minus four (*clpP, petL, rpl2*, and *rpl32*) that were problematic to align), pairwise distance analyses were performed initially between 10 taxa (*Ginkgo, Cycas, Pinus, Podocarpus, Amborella, Nuphar, Nymphaea, Calycanthus, Ranunculus*, and *Acorus*) and *Welwitschia *using MEGA3.1 [77]. The 10 taxa were chosen to represent all of the major clades of extant non-gnetophtye seed plants and constituted reference Set A. Distances were calculated using both the LogDet (data not shown) and the Kimura two-parameter models, which gave very similar results. Default MEGA3.1 parameters were used in all calculations. Standard error for each distance was calculated via bootstrapping with 100 replicates. The Relative Divergence Factor was calculated by dividing the average distance calculated from *Welwitschia *to each non-gnetophytes by the average distance from comparisons among the 10 non-gnetophytes. We repeated these calculations using Kimura two-parameter distance and different subsets of the ten taxa as reference sets. For each of the five sets of calculations, we determined whether or not the difference between the two average distances was significant using the two-tailed t-test. In addition, we preformed Tajima's Relative Rate test, using MEGA 4.0 [78], on the 57 concatenated genes.

### Phylogenetic Inference

We added *Welwitschia *plastome genes to the alignment/data matrix of Leebens-Mack et al [79]. As mentioned above, four of the "standard" 61 genes were excluded from our analyses. The pteridophytes *Psilotum *and *Adiantum *were used to root the gymnosperm tree, although all analyses were run as and trees generated as "unrooted" topologies, i.e., we did not force *Psilotum *and *Adiantum *to any particular position on any tree. Bootstrap (BS) trees (100 heuristic replicates) were generated under Minimum Evolution (LogDet distances), Maximum Parsimony (MP), and Maximum Likelihood (ML), using a GTR + I + Γ model, optimizations using PAUP4.0b10 [80]. Individual MP analyses were conducted using branch and bound searches, always yielding a single most parsimonious tree identical in topology to the BS tree. Multiple individual ML heuristic searches were also conducted using estimation of all model parameters and obtaining starting trees by 10 replicates of random stepwise addition. Mr. Bayes 3.1 [81] was used, with default settings except as noted, to conduct a Bayesian analysis again using GTR + I + Γ (the model indicated by ModelTest [82] as the best fit to the data). Two analyses were run in parallel for 100,000 generations. After 6,000 generations, the standard deviation between the two hot chains was 0.000000 and remained at that level for the remainder of the run. Visual analysis using AWTY [83] also indicated that the chains had converged very early in the run. The first ten percent of the trees were discarded as "burn-in". PAUP4b10 was used to construct the consensus tree for the 901 trees retained. The Shimodaira-Hasegawa, or SH, Test [84] was implemented in PAUP4b10 using 1000 replicates and RELL approximation.

## Abbreviations

Anthophyte: hypothesized lineage composed of gnetophytes and angiosperms (and extinct taxa), BI: Bayesian Inference, bp: base pairs, BS: Booststrap, Conifer II: conifers are composed of two major clades, Pinaceae and the other families; the non-Pinaceae clade has been designated 'conifer II' or more recently Cupressophyta [60], Gnepine: hypothesized group composed of gnetophytes and Pinaceae, Gnetifer: hypothesized clade composed of gnetophytes as sister to Pinaceae + ConiferII, GRIMM: Genome rearrangement algorithms (genome rearrangements in mouse and man) [75], IR: Inverted Repeat (in plastid genomes usually a large, 10–25 kb, region that includes the rRNA genes), LBA: Long Branch Attraction (a phenomenon that confounds phylogenetic reconstruction as rapidly evolving lineages may share more characteristics due to chance rather than descent), LSC: Large Single Copy (a region of the plastid genome), ME: Minimum Evolution, ML: Maximum Likelihood, MP: Maximum Parsimony, ORF: Open Reading Frame, PP: Posterior Probability, RCA: Rolling Circle Amplification, SSC: Small Single Copy (a region of the plastid genome), WEMI: *Welwitschia mirabilis*

## Authors' contributions

SRM annotated the genome, performed most analyses, and wrote the initial draft as his undergraduate thesis; JVK and JLB generated the draft genome sequence and initial assembly; JLB also assisted with manuscript preparation; LAR conceived of the project, helped with finishing, generated the final assembly, performed phylogenetic work and relative rate tests, assisted with other analyses, and revised the thesis for submission.

## Supplementary Material

Additional File 1 A+T percentage of various genome compartments. Click here for file

Additional File 2 Codon usage and A+T bias of 3^rd ^position. Click here for file

Additional File 3 Calculation of Relative Divergence Factor based on reference set A Click here for file

Additional File 4 Calculation of Relative Divergence Factor based on reference set B Click here for file

Additional File 5 Calculation of Relative Divergence Factor based on reference set C Click here for file

Additional File 6 Calculation of Relative Divergence Factor based on reference set D Click here for file

Additional File 7 Calculation of Relative Divergence Factor based on reference set E Click here for file
