# Supplementary material for: The complete plastid genome sequence of Welwitschia mirabilis: an unusually compact plastome with accelerated divergence rates
Source: BMC Evol Biol. 2008 May 1;8:130. doi: 10.1186/1471-2148-8-130 (PMC2386820; doi:10.1186/1471-2148-8-130)
Supplement: Additional File 1 — A+T percentage of various genome compartments. [file 1471-2148-8-130-S1.doc]

Supplemental Table 1. A+T percentages in the *Welwitschia mirabilis* plastid genome.

| Genomic Region | A+T % |
| --- | --- |
| Plastome, overall | 62 |
| Inverted Repeats | 58 |
| Large Single Copy Region | 67 |
| Small Single Copy Region | 68 |
| Coding regions | 63 |
| Protein coding genes | 77 |
| rRNA genes | 47 |
| tRNA genes | 47 |
| Non-coding regions | 66 |
| Introns | 61 |
| Intergenic Spacers | 67 |

The A+T content was performed using GEECEE: http://bioweb.pasteur.fr/seqanal/interfaces/geecee.html

Burskiewich, unpublished
