# Supplementary material for: The complete plastid genome sequence of Welwitschia mirabilis: an unusually compact plastome with accelerated divergence rates
Source: BMC Evol Biol. 2008 May 1;8:130. doi: 10.1186/1471-2148-8-130 (PMC2386820; doi:10.1186/1471-2148-8-130)
Supplement: Additional File 2 — Codon usage and A+T bias of 3rd position. [file 1471-2148-8-130-S2.doc]

**Supplemental Table 2. Codon usage in the *Welwitschia mirabilis* plastome.**

| **A.A.** | **Codon** | **Number** | **% A+T in**  **3rd Position** | **A.A.** | **Codon** | **Number** | **% A+T in**  **3rd Position** |
| --- | --- | --- | --- | --- | --- | --- | --- |
| Ala | GCG | 86 | 80.41% | Pro | CCG | 118 | 70.76% |
| Ala | GCA | 296 |  | Pro | CCA | 228 |  |
| Ala | GCT | 525 |  | Pro | CCT | 360 |  |
| Ala | GCC | 114 |  | Pro | CCC | 125 |  |
| Cys | TGT | 169 | 80.86% | Gln | CAG | 144 | 80.98% |
| Cys | TGC | 40 |  | Gln | CAA | 613 |  |
| Asp | GAT | 577 | 77.97% | Arg | AGG | 128 | 76.06% |
| Asp | GAC | 163 |  | Arg | AGA | 351 |  |
| Glu | GAG | 277 | 74.75% | Arg | CGG | 72 |  |
| Glu | GAA | 820 |  | Arg | CGA | 265 |  |
| Phe | TTT | 941 | 74.15% | Arg | CGT | 267 |  |
| Phe | TTC | 328 |  | Arg | CGC | 78 |  |
| Gly | GGG | 135 | 79.06% | Ser | AGT | 279 | 71.06% |
| Gly | GGA | 459 |  | Ser | AGC | 84 |  |
| Gly | GGT | 432 |  | Ser | TCG | 123 |  |
| Gly | GGC | 101 |  | Ser | TCA | 183 |  |
| His | CAT | 333 | 77.80% | Ser | TCT | 449 |  |
| His | CAC | 95 |  | Ser | TCC | 164 |  |
| Ile | ATA | 504 | 83.56% | Thr | ACG | 93 | 76.41% |
| Ile | ATT | 767 |  | Thr | ACA | 295 |  |
| Ile | ATC | 250 |  | Thr | ACT | 424 |  |
| Lys | AAG | 307 | 79.20% | Thr | ACC | 129 |  |
| Lys | AAA | 1169 |  | Val | GTG | 135 | 70.76% |
| Leu | TTG | 411 | 69.27% | Val | GTA | 397 |  |
| Leu | TTA | 692 |  | Val | GTT | 436 |  |
| Leu | CTG | 113 |  | Val | GTC | 106 |  |
| Leu | CTA | 254 |  | Trp | TGG | 355 | --- |
| Leu | CTT | 449 |  | Tyr | TAT | 523 | --- |
| Leu | CTC | 95 |  | Tyr | TAC | 134 | 79.60% |
| Met | ATG | 356 | --- | End | TGA | 13 | 87.69% |
| Asn | AAT | 659 | 74.04% | End | TAG | 8 |  |
| Asn | AAC | 231 |  | End | TAA | 44 |  |

A concatenated file of protein-coding sequences (all was generated: the gene sequences were extracted using DOGMA), one copy of any gene duplicated due to its inclusion in the inverted repeat was removed, all intervening textural material was deleted. The concatenated sequence was then submitted to the SMS codon usage calculator <http://www.bioinformatics.vg/sms/codon_usage.html> (Stothard, P. 2000. The sequence manipulation suite: JavaScript programs for analyzing and formatting protein and DNA sequences. *Biotechniques* **28**: 1102-1104.
