# Supplementary material for: The complete plastid genome sequence of Welwitschia mirabilis: an unusually compact plastome with accelerated divergence rates
Source: BMC Evol Biol. 2008 May 1;8:130. doi: 10.1186/1471-2148-8-130 (PMC2386820; doi:10.1186/1471-2148-8-130)
Supplement: Additional File 3 — Calculation of Relative Divergence Factor based on reference set A [file 1471-2148-8-130-S3.doc]

Supplemental Table 3. Relative Divergence Factor calculations for Set A.

| Gene | 10 Taxa  Average | SE | WEMI-  10 Taxa | SE | wemi  factor | t-score | p |
| --- | --- | --- | --- | --- | --- | --- | --- |
| All | 0.15655 | 0.010136 | 0.26456 | 0.00264 | 1.69 | 10.31 | **** |
| *atpA* | 0.14132 | 0.00652 | 0.21617 | 0.01147 | 1.53 | 5.67 | **** |
| *atpB* | 0.11617 | 0.00592 | 0.18278 | 0.01072 | 1.57 | 5.44 | **** |
| *atpE* | 0.19998 | 0.01598 | 0.31823 | 0.02862 | 1.59 | 3.61 | **** |
| *atpF* | 0.20972 | 0.01368 | 0.33113 | 0.02435 | 1.58 | 4.35 | **** |
| *atpH* | 0.10523 | 0.01605 | 0.14752 | 0.02328 | 1.40 | 1.50 | NS |
| *atpI* | 0.14162 | 0.00944 | 0.22703 | 0.01595 | 1.60 | 4.61 | **** |
| *ccsA* | 0.2554 | 0.01103 | 0.42917 | 0.02339 | 1.68 | 6.72 | **** |
| *cemA* | 0.24025 | 0.01325 | 0.36284 | 0.0237 | 1.51 | 4.51 | **** |
| *matK* | 0.37052 | 0.01229 | 0.70997 | 0.05112 | 1.92 | 6.46 | **** |
| *petA* | 0.1586 | 0.00888 | 0.26019 | 0.0161 | 1.64 | 5.53 | **** |
| *petB* | 0.09692 | 0.00788 | 0.16902 | 0.01567 | 1.74 | 4.11 | **** |
| *petD* | 0.11194 | 0.00964 | 0.18647 | 0.01808 | 1.67 | 3.64 | **** |
| *petG* | 0.12387 | 0.02317 | 0.2243 | 0.04464 | 1.81 | 2.00 | NS |
| *petN* | 0.0856 | 0.03625 | 0.11503 | 0.03215 | 1.34 | 0.61 | NS |
| *psaA* | 0.0856 | 0.01957 | 0.1667 | 0.00743 | 1.95 | 3.87 | **** |
| *psaB* | 0.10521 | 0.0048 | 0.16086 | 0.00796 | 1.53 | 5.99 | **** |
| *psaC* | 0.09798 | 0.01317 | 0.17645 | 0.02703 | 1.80 | 2.61 | * |
| *psaI* | 0.21545 | 0.03713 | 0.54735 | 0.11372 | 2.54 | 2.77 | ** |
| *psaJ* | 0.19148 | 0.0258 | 0.21578 | 0.03381 | 1.13 | 0.57 | NS |
| *psbA* | 0.09581 | 0.00582 | 0.14485 | 0.00993 | 1.51 | 4.26 | **** |
| *psbB* | 0.11319 | 0.00591 | 0.18324 | 0.00985 | 1.62 | 6.10 | **** |
| *psbC* | 0.10187 | 0.00599 | 0.16691 | 0.01093 | 1.64 | 5.22 | **** |
| *psbD* | 0.08625 | 0.00589 | 0.1519 | 0.01057 | 1.76 | 5.43 | **** |
| *psbE* | 0.12197 | 0.01489 | 0.1894 | 0.0264 | 1.55 | 2.22 | * |
| *psbF* | 0.07127 | 0.01473 | 0.21161 | 0.04667 | 2.97 | 2.87 | ** |
| *psbH* | 0.18636 | 0.02045 | 0.30811 | 0.03832 | 1.65 | 2.80 | ** |
| *psbI* | 0.1617 | 0.02634 | 0.22144 | 0.04166 | 1.37 | 1.21 | NS |
| *psbJ* | 0.13532 | 0.02364 | 0.29303 | 0.05405 | 2.17 | 2.67 | * |
| *psbK* | 0.23334 | 0.02709 | 0.3584 | 0.04682 | 1.54 | 2.31 | * |
| *psbL* | 0.0889 | 0.01802 | 0.11649 | 0.0279 | 1.31 | 0.83 | NS |
| *psbM* | 0.15217 | 0.02589 | 0.20213 | 0.04246 | 1.33 | 1.00 | NS |
| *psbN* | 0.11791 | 0.02081 | 0.21032 | 0.04123 | 1.78 | 2.00 | NS |
| *psbT* | 0.12575 | 0.02683 | 0.15766 | 0.03583 | 1.25 | 0.71 | NS |
| *psbZ* | 0.13048 | 0.01909 | 0.26556 | 0.03818 | 2.04 | 3.16 | *** |
| *rbcL* | 0.10753 | 0.00556 | 0.15896 | 0.00972 | 1.48 | 4.59 | **** |
| *rpl14* | 0.1587 | 0.01438 | 0.29449 | 0.02975 | 1.86 | 4.11 | **** |
| *rpl16* | 0.14193 | 0.01289 | 0.30335 | 0.02794 | 2.14 | 5.25 | **** |
| *rpl20* | 0.24077 | 0.01819 | 0.38703 | 0.03369 | 1.61 | 3.82 | **** |
| *rpl33* | 0.20139 | 0.0225 | 0.40646 | 0.04948 | 2.02 | 3.77 | **** |
| *rpl36* | 0.15044 | 0.02442 | 0.34017 | 0.06121 | 2.26 | 2.88 | ** |
| *rpoA* | 0.23598 | 0.01079 | 0.50783 | 0.02788 | 2.15 | 9.09 | **** |
| *rpoB* | 0.18432 | 0.00522 | 0.34796 | 0.01337 | 1.89 | 11.40 | **** |
| *rpoC1* | 0.18789 | 0.00708 | 0.38909 | 0.01601 | 2.07 | 11.49 | **** |
| *rpoC2* | 0.24724 | 0.00669 | 0.428 | 0.01298 | 1.73 | 12.38 | **** |
| *rps11* | 0.16943 | 0.01409 | 0.4433 | 0.03937 | 2.62 | 6.55 | **** |
| *rps12* | 0.07046 | 0.00948 | 0.13936 | 0.01844 | 1.98 | 3.32 | *** |
| *rps14* | 0.18671 | 0.0101 | 0.37641 | 0.03793 | 2.02 | 4.83 | **** |
| *rps15* | 0.31107 | 0.02572 | 0.58489 | 0.06183 | 1.88 | 4.09 | **** |
| *rps18* | 0.14087 | 0.01584 | 0.42112 | 0.05193 | 2.99 | 5.16 | **** |
| *rps19* | 0.17817 | 0.01665 | 0.3472 | 0.03567 | 1.95 | 4.29 | **** |
| *rps2* | 0.20287 | 0.01087 | 0.42614 | 0.0266 | 2.10 | 7.77 | **** |
| *rps3* | 0.22071 | 0.01436 | 0.48086 | 0.03164 | 2.18 | 7.49 | **** |
| *rps4* | 0.19485 | 0.01264 | 0.35585 | 0.0271 | 1.83 | 5.38 | **** |
| *rps7* | 0.08574 | 0.00865 | 0.18845 | 0.01973 | 2.20 | 4.77 | **** |
| *rps8* | 0.24934 | 0.01742 | 0.39186 | 0.03443 | 1.57 | 3.69 | **** |
| *ycf3* | 0.11351 | 0.00976 | 0.22085 | 0.0192 | 1.95 | 4.98 | **** |
| *ycf4* | 0.19463 | 0.01288 | 0.31702 | 0.02263 | 1.63 | 4.70 | **** |

An average distance was calculated for the reference taxa using Kimura two-parameter distance for those data shown here, but calculations based on LogDet distances were very similar. The reference taxa, for Set A, were *Ginkgo, Cycas, Pinus, Podocarpus, Amborella, Nuphar, Nymphaea, Calycanthus, Ranunculus,* and *Acorus.* Then an average pairwise distance of the *Welwitschia* sequence to the reference taxa was calculated. The ratio of the *Welwitschia* distance to the non-gnetophyte average is presented as the “wemi factor”. The *Welwitschia* average distance was compared to the non-gnetophyte average using the t-test to determine if the difference between the two means is significantly different. In the column headed ‘p’, symbols are used to indicate level of significance in the two-tailed t test: NS = not significant, * = p < 0.05, ** = p < 0.01, *** = p < 0.005, **** = p < 0.001.
