# Supplementary material for: The complete plastid genome sequence of Welwitschia mirabilis: an unusually compact plastome with accelerated divergence rates
Source: BMC Evol Biol. 2008 May 1;8:130. doi: 10.1186/1471-2148-8-130 (PMC2386820; doi:10.1186/1471-2148-8-130)
Supplement: Additional File 4 — Calculation of Relative Divergence Factor based on reference set B [file 1471-2148-8-130-S4.doc]

Supplemental Table 4. Relative Divergence Factor calculations for Set B.

| Gene | 4 taxa | std error | 4+WEMI | std error | WEMI FACTOR | t score | p |
| --- | --- | --- | --- | --- | --- | --- | --- |
| All | 0.16694 | 0.00232 | 0.25606 | 0.00222 | 1.53 | 27.75 | NS |
| *atpA* | 0.14858 | 0.00829 | 0.20177 | 0.01128 | 1.36 | 3.80 | ** |
| *atpB* | 0.12118 | 0.0077 | 0.17504 | 0.01016 | 1.44 | 4.22 | *** |
| *atpE* | 0.19915 | 0.01804 | 0.30294 | 0.02683 | 1.52 | 3.21 | * |
| *atpF* | 0.23478 | 0.01764 | 0.31021 | 0.02543 | 1.32 | 2.44 | * |
| *atpH* | 0.10029 | 0.01577 | 0.1361 | 0.02382 | 1.36 | 1.25 | NS |
| *atpI* | 0.13775 | 0.01031 | 0.21482 | 0.01574 | 1.56 | 4.10 | *** |
| *ccsA* | 0.25658 | 0.01449 | 0.39607 | 0.02247 | 1.54 | 5.22 | **** |
| *cemA* | 0.25049 | 0.0153 | 0.31777 | 0.02074 | 1.27 | 2.61 | * |
| *matK* | 0.38887 | 0.01475 | 0.68918 | 0.04717 | 1.77 | 6.08 | **** |
| *petA* | 0.16721 | 0.01067 | 0.24756 | 0.01499 | 1.48 | 4.37 | *** |
| *petB* | 0.08923 | 0.00886 | 0.15963 | 0.01574 | 1.79 | 3.90 | *** |
| *petD* | 0.10772 | 0.01193 | 0.16418 | 0.01694 | 1.52 | 2.72 | * |
| *petG* | 0.12918 | 0.02754 | 0.20078 | 0.04237 | 1.55 | 1.42 | NS |
| *petN* | 0.10917 | 0.0292 | 0.12643 | 0.03395 | 1.16 | 0.39 | NS |
| *psaA* | 0.11322 | 0.00571 | 0.15312 | 0.00785 | 1.35 | 4.11 | *** |
| *psaB* | 0.11272 | 0.00543 | 0.14981 | 0.00747 | 1.33 | 4.02 | *** |
| *psaC* | 0.08663 | 0.01516 | 0.16088 | 0.02476 | 1.86 | 2.56 | * |
| *psaI* | 0.27206 | 0.05118 | 0.55109 | 0.11238 | 2.03 | 2.26 | NS |
| *psaJ* | 0.25322 | 0.03633 | 0.22084 | 0.03419 | 0.87 | -0.65 | NS |
| *psbA* | 0.10548 | 0.00797 | 0.13943 | 0.01026 | 1.32 | 2.61 | * |
| *psbB* | 0.11061 | 0.00623 | 0.1736 | 0.00938 | 1.57 | 5.59 | **** |
| *psbC* | 0.10475 | 0.00701 | 0.1585 | 0.01031 | 1.51 | 4.31 | *** |
| *psbD* | 0.08493 | 0.00683 | 0.1423 | 0.01007 | 1.68 | 4.71 | *** |
| *psbE* | 0.12669 | 0.01886 | 0.17559 | 0.02349 | 1.39 | 1.62 | NS |
| *psbF* | 0.07275 | 0.01895 | 0.1917 | 0.04186 | 2.64 | 2.59 | * |
| *psbH* | 0.18688 | 0.02229 | 0.30161 | 0.03833 | 1.61 | 2.59 | * |
| *psbI* | 0.14523 | 0.02983 | 0.21087 | 0.04043 | 1.45 | 1.31 | NS |
| *psbJ* | 0.12496 | 0.02557 | 0.25665 | 0.04848 | 2.05 | 2.40 | * |
| *psbK* | 0.28005 | 0.03521 | 0.36345 | 0.04908 | 1.30 | 1.38 | NS |
| *psbL* | 0.11261 | 0.02522 | 0.1097 | 0.0263 | 0.97 | -0.08 | NS |
| *psbM* | 0.17668 | 0.0348 | 0.18933 | 0.03893 | 1.07 | 0.24 | NS |
| *psbN* | 0.14687 | 0.02974 | 0.19435 | 0.03685 | 1.32 | 1.00 | NS |
| *psbT* | 0.10642 | 0.02835 | 0.14752 | 0.03647 | 1.39 | 0.89 | NS |
| *psbZ* | 0.12303 | 0.02107 | 0.24847 | 0.03799 | 2.02 | 2.89 | * |
| *rbcL* | 0.10327 | 0.00602 | 0.14654 | 0.00968 | 1.42 | 3.80 | ** |
| *rpl14* | 0.16194 | 0.017 | 0.26306 | 0.02741 | 1.62 | 3.14 | * |
| *rpl16* | 0.13058 | 0.01422 | 0.29684 | 0.02741 | 2.27 | 5.38 | **** |
| *rpl20* | 0.2479 | 0.02192 | 0.38001 | 0.03449 | 1.53 | 3.23 | * |
| *rpl33* | 0.16397 | 0.02557 | 0.39129 | 0.05557 | 2.39 | 3.72 | ** |
| *rpl36* | 0.16075 | 0.03418 | 0.31989 | 0.06057 | 1.99 | 2.29 | NS |
| *rpoA* | 0.21201 | 0.01298 | 0.48034 | 0.02947 | 2.27 | 8.33 | **** |
| *rpoB* | 0.18888 | 0.00634 | 0.31968 | 0.01094 | 1.69 | 10.34 | **** |
| *rpoC1* | 0.22537 | 0.01021 | 0.38083 | 0.01568 | 1.69 | 8.31 | **** |
| *rpoC2* | 0.24331 | 0.00808 | 0.38365 | 0.01256 | 1.58 | 9.40 | **** |
| *rps11* | 0.15092 | 0.01644 | 0.39032 | 0.03873 | 2.59 | 5.69 | **** |
| *rps12* | 0.05935 | 0.00956 | 0.102011 | 0.01699 | 1.72 | 2.19 | NS |
| *rps14* | 0.17471 | 0.02036 | 0.35052 | 0.03847 | 2.01 | 4.04 | *** |
| *rps15* | 0.3153 | 0.03718 | 0.542 | 0.06491 | 1.72 | 3.03 | * |
| *rps18* | 0.21585 | 0.02595 | 0.46312 | 0.05365 | 2.15 | 4.15 | *** |
| *rps19* | 0.16077 | 0.01879 | 0.35134 | 0.03872 | 2.19 | 4.43 | *** |
| *rps2* | 0.19805 | 0.01318 | 0.39982 | 0.02587 | 2.02 | 6.95 | **** |
| *rps3* | 0.22232 | 0.01462 | 0.48891 | 0.03132 | 2.20 | 7.71 | **** |
| *rps4* | 0.20524 | 0.01379 | 0.35479 | 0.02626 | 1.73 | 5.04 | **** |
| *rps7* | 0.08071 | 0.0093 | 0.19109 | 0.01934 | 2.37 | 5.14 | **** |
| *rps8* | 0.26846 | 0.02069 | 0.34641 | 0.02914 | 1.29 | 2.18 | NS |
| *ycf3* | 0.12453 | 0.01174 | 0.19064 | 0.01791 | 1.53 | 3.09 | * |
| *ycf4* | 0.20241 | 0.01505 | 0.27477 | 0.01998 | 1.36 | 2.89 | * |
