# Supplementary material for: The complete plastid genome sequence of Welwitschia mirabilis: an unusually compact plastome with accelerated divergence rates
Source: BMC Evol Biol. 2008 May 1;8:130. doi: 10.1186/1471-2148-8-130 (PMC2386820; doi:10.1186/1471-2148-8-130)
Supplement: Additional File 5 — Calculation of Relative Divergence Factor based on reference set C [file 1471-2148-8-130-S5.doc]

Supplemental Table 5. Relative Divergence Factor calculations for Set C.

| Gene | 4 Taxon Average | SE | Wemi –  4 taxa | SE | WEMI FACTOR | t score | p |
| --- | --- | --- | --- | --- | --- | --- | --- |
| All | 0.15598 | 0.00108 | 0.25976 | 0.00202 | 1.67 | 45.31 | **** |
| *atpA* | 0.13659 | 0.00754 | 0.19675 | 0.01119 | 1.44 | 4.46 | *** |
| *atpB* | 0.10922 | 0.00735 | 0.17315 | 0.01 | 1.59 | 5.15 | **** |
| *atpE* | 0.18778 | 0.0176 | 0.30608 | 0.02724 | 1.63 | 3.65 | ** |
| *atpF* | 0.21806 | 0.01694 | 0.30345 | 0.02205 | 1.39 | 3.07 | * |
| *atpH* | 0.10883 | 0.01814 | 0.13371 | 0.02389 | 1.23 | 0.83 | NS |
| *atpI* | 0.13477 | 0.00996 | 0.22094 | 0.01781 | 1.64 | 4.22 | *** |
| *ccsA* | 0.22771 | 0.01326 | 0.41129 | 0.02134 | 1.81 | 7.31 | **** |
| *cemA* | 0.21008 | 0.01388 | 0.31764 | 0.02262 | 1.51 | 4.05 | *** |
| *matK* | 0.35844 | 0.01559 | 0.68286 | 0.05278 | 1.91 | 5.89 | **** |
| *petA* | 0.13846 | 0.00962 | 0.23228 | 0.01451 | 1.68 | 5.39 | **** |
| *petB* | 0.08706 | 0.00839 | 0.15964 | 0.01515 | 1.83 | 4.19 | *** |
| *petD* | 0.10064 | 0.01057 | 0.16628 | 0.01777 | 1.65 | 3.17 | * |
| *petG* | 0.09979 | 0.02378 | 0.19412 | 0.04319 | 1.95 | 1.91 | NS |
| *petN* | 0.09964 | 0.02711 | 0.11586 | 0.03225 | 1.16 | 0.38 | NS |
| *psaA* | 0.10026 | 0.00525 | 0.1528 | 0.00709 | 1.52 | 5.96 | **** |
| *psaB* | 0.10335 | 0.00502 | 0.14643 | 0.00775 | 1.42 | 4.67 | *** |
| *psaC* | 0.08563 | 0.0147 | 0.16769 | 0.0263 | 1.96 | 2.72 | * |
| *psaI* | 0.2431 | 0.04529 | 0.55219 | 0.01163 | 2.27 | 6.61 | **** |
| *psaJ* | 0.23781 | 0.03523 | 0.22162 | 0.03607 | 0.93 | -0.32 | NS |
| *psbA* | 0.10331 | 0.00805 | 0.14307 | 0.01039 | 1.38 | 3.03 | * |
| *psbB* | 0.10398 | 0.0054 | 0.17279 | 0.00901 | 1.66 | 6.55 | **** |
| *psbC* | 0.10288 | 0.00715 | 0.15428 | 0.01022 | 1.50 | 4.12 | *** |
| *psbD* | 0.08456 | 0.00662 | 0.14086 | 0.01017 | 1.67 | 4.64 | *** |
| *psbE* | 0.10132 | 0.016 | 0.1763 | 0.02641 | 1.74 | 2.43 | * |
| *psbF* | 0.05557 | 0.01603 | 0.19186 | 0.04562 | 3.45 | 2.82 | * |
| *psbH* | 0.18383 | 0.02353 | 0.2888 | 0.03699 | 1.57 | 2.39 | * |
| *psbI* | 0.14439 | 0.02878 | 0.20679 | 0.04295 | 1.43 | 1.21 | NS |
| *psbJ* | 0.12496 | 0.02475 | 0.26753 | 0.049 | 2.14 | 2.60 | * |
| *psbK* | 0.26852 | 0.03309 | 0.36136 | 0.04721 | 1.35 | 1.61 | NS |
| *psbL* | 0.1089 | 0.02368 | 0.11785 | 0.02649 | 1.08 | 0.25 | NS |
| *psbM* | 0.15157 | 0.03407 | 0.20662 | 0.04152 | 1.36 | 1.02 | NS |
| *psbN* | 0.13138 | 0.02675 | 0.19726 | 0.03812 | 1.50 | 1.41 | NS |
| *psbT* | 0.08253 | 0.02427 | 0.14455 | 0.04073 | 1.75 | 1.31 | NS |
| *psbZ* | 0.11048 | 0.01871 | 0.2415 | 0.03656 | 2.19 | 3.19 | * |
| *rbcL* | 0.0903 | 0.00572 | 0.14548 | 0.00949 | 1.61 | 4.98 | *** |
| *rpl14* | 0.16817 | 0.01686 | 0.27271 | 0.02678 | 1.62 | 3.30 | * |
| *rpl16* | 0.12998 | 0.01413 | 0.29595 | 0.02697 | 2.28 | 5.45 | **** |
| *rpl20* | 0.2435 | 0.02163 | 0.37423 | 0.03399 | 1.54 | 3.24 | * |
| *rpl33* | 0.16942 | 0.02755 | 0.39317 | 0.0496 | 2.32 | 3.94 | *** |
| *rpl36* | 0.13497 | 0.0287 | 0.30817 | 0.05616 | 2.28 | 2.75 | * |
| *rpoA* | 0.21392 | 0.01359 | 0.51378 | 0.02843 | 2.40 | 9.52 | **** |
| *rpoB* | 0.17507 | 0.00608 | 0.31888 | 0.01088 | 1.82 | 11.54 | **** |
| *rpoC1* | 0.21066 | 0.00973 | 0.37197 | 0.01428 | 1.77 | 9.34 | **** |
| *rpoC2* | 0.23469 | 0.00744 | 0.398992 | 0.01106 | 1.70 | 12.33 | **** |
| *rps11* | 0.13525 | 0.01376 | 0.38358 | 0.03901 | 2.84 | 6.00 | **** |
| *rps12* | 0.08635 | 0.01217 | 0.12607 | 0.01794 | 1.46 | 1.83 | NS |
| *rps14* | 0.15545 | 0.01726 | 0.366 | 0.03735 | 2.35 | 5.12 | **** |
| *rps15* | 0.37812 | 0.03552 | 0.54104 | 0.06057 | 1.43 | 2.32 | * |
| *rps18* | 0.2034 | 0.02397 | 0.4947 | 0.05815 | 2.43 | 4.63 | *** |
| *rps19* | 0.17108 | 0.01928 | 0.35411 | 0.03736 | 2.07 | 4.35 | *** |
| *rps2* | 0.19278 | 0.01272 | 0.39393 | 0.02608 | 2.04 | 6.93 | **** |
| *rps3* | 0.21098 | 0.01458 | 0.49571 | 0.03522 | 2.35 | 7.47 | **** |
| *rps4* | 0.20196 | 0.01554 | 0.35538 | 0.02403 | 1.76 | 5.36 | **** |
| *rps7* | 0.0744 | 0.00936 | 0.1917 | 0.0199 | 2.58 | 5.33 | **** |
| *rps8* | 0.24933 | 0.01956 | 0.3482 | 0.02956 | 1.40 | 2.79 | * |
| *ycf3* | 0.12764 | 0.01163 | 0.19648 | 0.01706 | 1.54 | 3.33 | * |
| *ycf4* | 0.18006 | 0.01374 | 0.26836 | 0.02041 | 1.49 | 3.59 | ** |
