# Supplementary material for: The complete plastid genome sequence of Welwitschia mirabilis: an unusually compact plastome with accelerated divergence rates
Source: BMC Evol Biol. 2008 May 1;8:130. doi: 10.1186/1471-2148-8-130 (PMC2386820; doi:10.1186/1471-2148-8-130)
Supplement: Additional File 6 — Calculation of Relative Divergence Factor based on reference set D [file 1471-2148-8-130-S6.doc]

Supplemental Table 4. Relative Divergence Factor calculations for Set D.

| Gene | 4 taxon average | SE | WEMI to 4 taxa | SE | WEMI FACTOR | t score | p |
| --- | --- | --- | --- | --- | --- | --- | --- |
| All | 0.17097 | 0.00122 | 0.25587 | 0.00174 | 1.50 | 46.91 | **** |
| *atpA* | 0.15792 | 0.00821 | 0.2051 | 0.01143 | 1.30 | 3.96 | *** |
| *atpB* | 0.13006 | 0.00746 | 0.17798 | 0.00994 | 1.37 | 4.61 | *** |
| *atpE* | 0.20644 | 0.01766 | 0.29724 | 0.02757 | 1.44 | 3.19 | * |
| *atpF* | 0.23625 | 0.01778 | 0.30698 | 0.02397 | 1.30 | 2.82 | * |
| *atpH* | 0.09684 | 0.01561 | 0.1372 | 0.02162 | 1.42 | 1.79 | NS |
| *atpI* | 0.15422 | 0.01154 | 0.22357 | 0.01627 | 1.45 | 4.09 | *** |
| *ccsA* | 0.26498 | 0.01498 | 0.39507 | 0.02262 | 1.49 | 5.55 | **** |
| *cemA* | 0.26653 | 0.01662 | 0.32605 | 0.02135 | 1.22 | 2.66 | * |
| *matK* | 0.41278 | 0.02879 | 0.7114 | 0.05069 | 1.72 | 5.74 | **** |
| *petA* | 0.17221 | 0.01059 | 0.24633 | 0.01562 | 1.43 | 4.57 | *** |
| *petB* | 0.09663 | 0.00901 | 0.16045 | 0.01546 | 1.66 | 4.02 | *** |
| *petD* | 0.1068 | 0.01176 | 0.16464 | 0.01773 | 1.54 | 3.15 | * |
| *petG* | 0.13496 | 0.02869 | 0.20078 | 0.03951 | 1.49 | 1.60 | NS |
| *petN* | 0.10419 | 0.02599 | 0.11279 | 0.0308 | 1.08 | 0.26 | NS |
| *psaA* | 0.11322 | 0.00603 | 0.15508 | 0.00836 | 1.37 | 4.80 | *** |
| *psaB* | 0.1126 | 0.00548 | 0.15369 | 0.00791 | 1.36 | 5.00 | *** |
| *psaC* | 0.08663 | 0.01499 | 0.15314 | 0.02347 | 1.77 | 2.74 | * |
| *psaI* | 0.27167 | 0.04641 | 0.54978 | 0.11133 | 2.02 | 2.46 | * |
| *psaJ* | 0.18641 | 0.03011 | 0.1948 | 0.03737 | 1.05 | 0.21 | NS |
| *psbA* | 0.10666 | 0.00797 | 0.13769 | 0.01046 | 1.29 | 2.83 | * |
| *psbB* | 0.11491 | 0.00594 | 0.1714 | 0.00896 | 1.49 | 6.09 | **** |
| *psbC* | 0.10612 | 0.00748 | 0.1618 | 0.01066 | 1.52 | 5.02 | *** |
| *psbD* | 0.09047 | 0.00677 | 0.13219 | 0.01043 | 1.46 | 3.87 | *** |
| *psbE* | 0.14367 | 0.01891 | 0.17903 | 0.02313 | 1.25 | 1.45 | NS |
| *psbF* | 0.06641 | 0.01788 | 0.19719 | 0.04531 | 2.97 | 2.85 | * |
| *psbH* | 0.21402 | 0.02569 | 0.29799 | 0.03692 | 1.39 | 2.19 | NS |
| *psbI* | 0.16974 | 0.03342 | 0.20804 | 0.04093 | 1.23 | 0.89 | NS |
| *psbJ* | 0.14222 | 0.02786 | 0.25341 | 0.04899 | 1.78 | 2.21 | NS |
| *psbK* | 0.26584 | 0.03452 | 0.33166 | 0.0447 | 1.25 | 1.40 | NS |
| *psbL* | 0.09031 | 0.02189 | 0.1072 | 0.02896 | 1.19 | 0.56 | NS |
| *psbM* | 0.15031 | 0.03026 | 0.15374 | 0.0345 | 1.02 | 0.09 | NS |
| *psbN* | 0.14852 | 0.02864 | 0.1913 | 0.03566 | 1.29 | 1.14 | NS |
| *psbT* | 0.1895 | 0.03807 | 0.1774 | 0.03628 | 0.94 | -0.31 | NS |
| *psbZ* | 0.1355 | 0.02164 | 0.25921 | 0.03821 | 1.91 | 3.15 | * |
| *rbcL* | 0.11407 | 0.00671 | 0.15076 | 0.00947 | 1.32 | 3.72 | ** |
| *rpl14* | 0.16413 | 0.0171 | 0.26968 | 0.02822 | 1.64 | 3.63 | ** |
| *rpl16* | 0.14614 | 0.01443 | 0.29575 | 0.02915 | 2.02 | 5.03 | *** |
| *rpl20* | 0.2546 | 0.0209 | 0.36935 | 0.03189 | 1.45 | 3.48 | ** |
| *rpl33* | 0.15517 | 0.02396 | 0.37767 | 0.05381 | 2.43 | 4.07 | *** |
| *rpl36* | 0.15541 | 0.03258 | 0.3268 | 0.06035 | 2.10 | 2.77 | * |
| *rpoA* | 0.25285 | 0.02355 | 0.487 | 0.02897 | 1.93 | 7.67 | **** |
| *rpoB* | 0.21696 | 0.00629 | 0.32309 | 0.01054 | 1.49 | 9.78 | **** |
| *rpoC1* | 0.24316 | 0.01032 | 0.27639 | 0.01697 | 1.14 | 1.90 | NS |
| *rpoC2* | 0.26897 | 0.00805 | 0.39392 | 0.01172 | 1.46 | 10.27 | **** |
| *rps11* | 0.17336 | 0.01815 | 0.38747 | 0.03773 | 2.24 | 5.57 | **** |
| *rps12* | 0.08919 | 0.01176 | 0.13329 | 0.01817 | 1.49 | 2.35 | * |
| *rps14* | 0.20194 | 0.02141 | 0.3586 | 0.03657 | 1.78 | 4.17 | *** |
| *rps15* | 0.34048 | 0.03785 | 0.54952 | 0.06691 | 1.61 | 3.04 | * |
| *rps18* | 0.20077 | 0.02404 | 0.48108 | 0.05614 | 2.40 | 4.92 | *** |
| *rps19* | 0.17186 | 0.01908 | 0.3447 | 0.03693 | 2.01 | 4.58 | *** |
| *rps2* | 0.22699 | 0.0151 | 0.41399 | 0.02614 | 1.82 | 6.96 | **** |
| *rps3* | 0.23649 | 0.01644 | 0.47795 | 0.03233 | 2.02 | 7.31 | **** |
| *rps4* | 0.19623 | 0.01508 | 0.33052 | 0.02563 | 1.68 | 5.09 | **** |
| *rps7* | 0.12399 | 0.01248 | 0.20342 | 0.02136 | 1.64 | 3.62 | ** |
| *rps8* | 0.28784 | 0.02266 | 0.35429 | 0.02884 | 1.23 | 2.19 | NS |
| *ycf3* | 0.12057 | 0.01174 | 0.20337 | 0.01748 | 1.69 | 4.57 | *** |
| *ycf4* | 0.21648 | 0.01579 | 0.28554 | 0.02191 | 1.32 | 3.02 | * |
