# Supplementary material for: The complete plastid genome sequence of Welwitschia mirabilis: an unusually compact plastome with accelerated divergence rates
Source: BMC Evol Biol. 2008 May 1;8:130. doi: 10.1186/1471-2148-8-130 (PMC2386820; doi:10.1186/1471-2148-8-130)
Supplement: Additional File 7 — Calculation of Relative Divergence Factor based on reference set E [file 1471-2148-8-130-S7.doc]

Supplemental Table 7. Relative Divergence Factor calculations for Set E.

| Gene | 5 taxon average | SE | WEMI – 5 taxa | std error (wemi) | WEMI Factor | t score | p |
| --- | --- | --- | --- | --- | --- | --- | --- |
| All | 0.16246 | 0.001 | 0.25049 | 0.00123 | 1.54 | 55.53 | **** |
| *atpA* | 0.15056 | 0.00726 | 0.19838 | 0.01122 | 1.32 | 3.58 | *** |
| *atpB* | 0.12754 | 0.00672 | 0.1764 | 0.00959 | 1.38 | 4.17 | *** |
| *atpE* | 0.19881 | 0.01662 | 0.29195 | 0.02576 | 1.47 | 3.04 | ** |
| *atpF* | 0.22882 | 0.01611 | 0.30332 | 0.02335 | 1.33 | 2.63 | * |
| *atpH* | 0.09892 | 0.01464 | 0.13628 | 0.02325 | 1.38 | 1.36 | NS |
| *atpI* | 0.14363 | 0.01032 | 0.21475 | 0.0154 | 1.50 | 3.84 | *** |
| *ccsA* | 0.24747 | 0.01324 | 0.39053 | 0.02326 | 1.58 | 5.35 | **** |
| *cemA* | 0.2461 | 0.01357 | 0.31286 | 0.02048 | 1.27 | 2.72 | * |
| *matK* | 0.38637 | 0.02341 | 0.70091 | 0.0509 | 1.81 | 5.61 | **** |
| *petA* | 0.16245 | 0.00927 | 0.24212 | 0.01496 | 1.49 | 4.53 | **** |
| *petB* | 0.09134 | 0.00855 | 0.15896 | 0.011516 | 1.74 | 4.71 | **** |
| *petD* | 0.10246 | 0.0105 | 0.1642 | 0.1642 | 1.60 | 0.38 | NS |
| *petG* | 0.12137 | 0.02472 | 0.20063 | 0.04033 | 1.65 | 1.68 | NS |
| *petN* | 0.10786 | 0.0251 | 0.11823 | 0.02941 | 1.10 | 0.27 | NS |
| *psaA* | 0.11021 | 0.00564 | 0.15221 | 0.00799 | 1.38 | 4.29 | **** |
| *psaB* | 0.10992 | 0.00511 | 0.15079 | 0.00747 | 1.37 | 4.52 | **** |
| *psaC* | 0.08052 | 0.01315 | 0.15678 | 0.02432 | 1.95 | 2.76 | * |
| *psaI* | 0.28457 | 0.04525 | 0.55526 | 0.11027 | 1.95 | 2.27 | * |
| *psaJ* | 0.23312 | 0.03397 | 0.2048 | 0.03365 | 0.88 | -0.59 | NS |
| *psbA* | 0.10319 | 0.00729 | 0.13827 | 0.01019 | 1.34 | 2.80 | * |
| *psbB* | 0.11151 | 0.00549 | 0.1703 | 0.00879 | 1.53 | 5.67 | **** |
| *psbC* | 0.10506 | 0.00671 | 0.15908 | 0.01041 | 1.51 | 4.36 | **** |
| *psbD* | 0.08416 | 0.00595 | 0.13949 | 0.01047 | 1.66 | 4.59 | **** |
| *psbE* | 0.13006 | 0.01693 | 0.17615 | 0.02488 | 1.35 | 1.53 | NS |
| *psbF* | 0.0719 | 0.01644 | 0.19226 | 0.04168 | 2.67 | 2.69 | * |
| *psbH* | 0.20341 | 0.02341 | 0.29236 | 0.03797 | 1.44 | 1.99 | NS |
| *psbI* | 0.15081 | 0.02713 | 0.20282 | 0.03989 | 1.34 | 1.08 | NS |
| *psbJ* | 0.13792 | 0.0261 | 0.25628 | 0.04914 | 1.86 | 2.13 | NS |
| *psbK* | 0.25441 | 0.03422 | 0.32834 | 0.04276 | 1.29 | 1.35 | NS |
| *psbL* | 0.10501 | 0.02137 | 0.10858 | 0.02769 | 1.03 | 0.10 | NS |
| *psbM* | 0.18032 | 0.02982 | 0.17776 | 0.03434 | 0.99 | -0.06 | NS |
| *psbN* | 0.14048 | 0.02634 | 0.18683 | 0.03678 | 1.33 | 1.02 | NS |
| *psbT* | 0.13489 | 0.02936 | 0.15619 | 0.03675 | 1.16 | 0.45 | NS |
| *psbZ* | 0.12463 | 0.02009 | 0.25109 | 0.03946 | 2.01 | 2.86 | * |
| *rbcL* | 0.10932 | 0.00584 | 0.14881 | 0.00924 | 1.36 | 3.61 | *** |
| *rpl14* | 0.15506 | 0.01595 | 0.25986 | 0.02632 | 1.68 | 3.41 | *** |
| *rpl16* | 0.1395 | 0.01365 | 0.29195 | 0.0275 | 2.09 | 4.97 | **** |
| *rpl20* | 0.23615 | 0.02021 | 0.36208 | 0.03285 | 1.53 | 3.27 | ** |
| *rpl33* | 0.15572 | 0.02449 | 0.38235 | 0.05428 | 2.46 | 3.81 | *** |
| *rpl36* | 0.17316 | 0.02966 | 0.3176 | 0.05637 | 1.83 | 2.27 | * |
| *rpoA* | 0.23626 | 0.01236 | 0.47866 | 0.02751 | 2.03 | 8.04 | **** |
| *rpoB* | 0.19868 | 0.0056 | 0.31436 | 0.01043 | 1.58 | 9.77 | **** |
| *rpoC1* | 0.23477 | 0.01046 | 0.367618 | 0.01741 | 1.57 | 6.54 | **** |
| *rpoC2* | 0.24591 | 0.0078 | 0.3779 | 0.01218 | 1.54 | 9.13 | **** |
| *rps11* | 0.16163 | 0.01632 | 0.38827 | 0.03805 | 2.40 | 5.47 | **** |
| *rps12* | 0.07952 | 0.01072 | 0.12742 | 0.01726 | 1.60 | 2.36 | * |
| *rps14* | 0.18208 | 0.01845 | 0.34815 | 0.03744 | 1.91 | 3.98 | *** |
| *rps15* | 0.31681 | 0.03336 | 0.53888 | 0.0666 | 1.70 | 2.98 | * |
| *rps18* | 0.21764 | 0.02436 | 0.45822 | 0.05434 | 2.11 | 4.04 | *** |
| *rps19* | 0.16698 | 0.01749 | 0.34253 | 0.03722 | 2.05 | 4.27 | **** |
| *rps2* | 0.21704 | 0.01209 | 0.40096 | 0.02507 | 1.85 | 6.61 | **** |
| *rps3* | 0.2244 | 0.01544 | 0.47057 | 0.03482 | 2.10 | 6.46 | **** |
| *rps4* | 0.21286 | 0.01425 | 0.34383 | 0.02398 | 1.62 | 4.70 | **** |
| *rps7* | 0.11813 | 0.01174 | 0.2 | 0.02028 | 1.69 | 3.49 | *** |
| *rps8* | 0.25936 | 0.01897 | 0.34169 | 0.029 | 1.32 | 2.38 | * |
| *ycf3* | 0.11576 | 0.01014 | 0.18843 | 0.01691 | 1.63 | 3.69 | *** |
| *ycf4* | 0.2075 | 0.01448 | 0.217519 | 0.02083 | 1.05 | 0.39 | NS |
